# Development and validation of a risk nomogram predicting pneumothorax requiring chest tube placement post-percutaneous CT-guided lung biopsy

**DOI:** 10.1186/s12880-025-01794-y

**Published:** 2025-07-01

**Authors:** Masha Bondarenko, Jianxiang Zhang, Ulysis Hugo Baal, Brian Lam, Gunvant Chaudhari, Yoo Jin Lee, Jamie Schroeder, Maya Vella, Brian Haas, Thienkhai Vu, Kimberly Kallianos, Jonathan Liu, Shravan Sridhar, Brett Elicker, Jae Ho Sohn

**Affiliations:** 1 Department of Radiology and Biomedical Imaging, University of California San Francisco (UCSF), 185 Berry Street, Suite 350, Lobby 6, San Francisco, CA 94107 USA; 2 University of California Berkeley (UCB), Berkeley, CA USA

**Keywords:** Pneumothorax, Image-guided biopsy, Lung neoplasms

## Abstract

**Background:**

Pneumothorax requiring chest tube after CT-guided transthoracic lung biopsy presents added clinical risk and costs to the healthcare system. Identifying high-risk patients can prompt alternative biopsy modes and/or better preparation for more focused post-procedural care. We aimed to develop and externally validate a risk nomogram for pneumothorax requiring chest tube placement following CT-guided lung biopsy, leveraging quantitative emphysema algorithm.

**Methods:**

This two-center retrospective study included patients who underwent CT-guided lung biopsy from between 1994 and 2023. Data from one hospital was set aside for validation (n = 613). Emphysema severity was quantified and categorized to 3-point scale using a previously published algorithm based on 3×3×3 kernels and Hounsfield thresholding, and a risk calculator was developed using forward variable selection and logistic regression. The model was validated using bootstrapping and Harrell’s C-index.

**Results:**

2,512 patients (mean age, 64.47 years ± 13.38 [standard deviation]; 1250 men) were evaluated, of whom 157 (6.7%) experienced pneumothorax complications requiring chest tube placement. After forward variable selection to reduce the covariates to maximize clinical usability, the risk score was developed using age over 60 (OR 1.80 [1.15–2.93]), non-prone patient position (OR 2.48 [1.63–3.75]), and severe emphysema (OR 1.99 [1.35–2.94]). The nomogram showed a mean absolute error of 0.5% in calibration and Harrell’s C-index of 0.664 in discrimination in the internal cohort.

**Conclusion:**

The developed nomogram predicts age over 60, non-prone position during biopsy, and severe emphysema to be most predictive of pneumothorax requiring chest tube placement following CT-guided lung biopsy.

**Supplementary Information:**

The online version contains supplementary material available at 10.1186/s12880-025-01794-y.

## Background

CT-guided transthoracic lung biopsy is a widely utilized procedure for diagnostic work up of pulmonary nodules and masses. Although it is considered safe and minimally invasive, the procedure can still be associated with complications, such as pneumothorax, hemothorax, and pulmonary hemorrhage [1, 2]. Among these complications, pneumothorax is the most common, reportedly occurring in 20–30% of cases [1, 3–15]. Larger pneumothoraces, occurring between 2–17% of procedures, may require chest tube placement, which is associated with increased healthcare costs and risk of hospital-related infections for patients [1, 6, 8, 9, 12, 15–18].

Clinical predictors for complications such as age, gender, patient position, and nodule location have been studied extensively in the literature. The risk associated with emphysema has been reported, although occasionally contested, especially in mild to moderate cases [1, 3, 4, 7, 8, 12, 16, 17, 19–23]. Those that have primarily utilized radiologist annotations to classify emphysema presence had significant variability and limited dataset size. Risk models using logistic regression have also been demonstrated in the literature as accurate tools to predict complication, but few have reliably and quantitatively utilized emphysema as a factor.

Some studies have also investigated the use of machine learning models to automatically and reliably classify emphysema extent for the prediction of lung biopsy complication, or directly used chest CT for predictions, but most models were ‘black box’ and/or difficult to translate in a wide variety of clinical settings due to excessive number of covariates [4, 16] Part of the debate and variability on identifying predictors of pneumothorax may be attributable to many studies having small sample sizes (<1000 patients) and training/validation data originating from a single institution, leading to reduced generalizability of models and results across hospitals.

There is a clear unmet need for a large, multi-institutionally validated algorithm that combines quantitative analysis with simplicity, enabling seamless integration into clinical practice. To address this, we developed and externally validated a risk calculator for pneumothorax complications requiring chest tube placement following CT-guided lung biopsy. Our approach incorporates a quantitative emphysema algorithm, with a strong emphasis on immediate clinical applicability.

## Methods

### Study population and biopsy protocols

This retrospective, institutional review board approved, informed consent waived, and Health Insurance and Portability Accountability Act compliant study included patients from two hospitals who underwent CT-guided lung biopsy with chest tube placement for pneumothorax management. Cases were from between February 1, 1994, and June 7, 2022, at University of California, San Francisco (UCSF) and between April 16, 2014 and January 30, 2023 at Zuckerberg San Francisco General Hospital (ZSFG). The two hospitals, although affiliated under a single academic institution, had distinct lung biopsy protocols, with mostly distinct primary procedural operators (less than 20% overlapping radiologists across two hospitals). UCSF is a tertiary academic referral center and ZSFG is a county safety net hospital. The patients at UCSF and ZSFG were mutually exclusive. Operators at UCSF utilized the breath hold technique upon needle entry without sedation and with variable use of autologous blood patch at the end of the procedure. ZSFG used free breathing technique upon needle entry under moderate sedation followed by saline patch at the end of the procedure. Both hospitals utilized a combination of fine needle aspiration and core biopsy techniques, with the number and type of samples varying based on patient tolerance, on-site confirmation of sample adequacy, and provider preference. The procedure has evolved over the time period assessed, but its fundamental aspects have remained consistent, as reflected in the variables subsequently analyzed.

Post-biopsy chest radiographs were obtained at the provider’s discretion, with one or two radiographs performed within two hours of the procedure during the patient observation period. These follow-up scans were evaluated against the immediate post-biopsy CT to detect any new or enlarging pneumothorax. Chest tube placement was determined by the provider’s discretion for moderate, enlarging, or symptomatic pneumothoraces. In certain cases, patients with smaller pneumothoraces who were unable to manage their care or reliably return to the emergency department were also preemptively treated with a chest tube. All chest tube insertions were conducted by the Interventional Radiology team, and patients were admitted overnight for observation.

The ground truth label for chest tube placement was determined semi-automatically by cross-referencing radiology reports and exam codes for chest tube placement with CT-guided lung biopsy reports. Manual confirmation involved reviewing radiograph reports for each biopsy procedure to verify whether an intervention was performed and to ensure that the pneumothorax was exclusively related to the biopsy procedure.

### Data preprocessing

Procedural covariates were compiled through report text extraction using both Excel and Python to search for key terms and their variations (e.g. “Right Middle Lobe”, “RML”, “middle lobe”). For cases where key details, such as patient positioning or nodule location, were unavailable in the reports, procedural CT scans were manually analyzed. Patient positioning was inferred under the assumption that the patient remained in the same position during both the scan and the biopsy procedure, while nodule location was determined by identifying and classifying the lobe in which the nodule was located directly from the CT scans. Primary reasons for persistent missing data was that CT scans were not accessible, the biopsy was either not a lung biopsy or was a lung biopsy that did not puncture the pleura (ie. biopsy of chest wall or mediastinum), and/or the report was missing. Data from ZSFG was put aside in model development and used for external validation of the logistic regression model.

### Quantitative emphysema algorithm

A previously developed and in-house quantitative emphysema algorithm was adapted to assess the degree and severity of emphysema in each patient from the biopsy CT scans. This algorithm, developed with an aim of user transparency, is based on CT image analysis, which uses 3×3×3 kernels and Hounsfield Unit thresholding to determine the extent of emphysema in the lung parenchyma [24]. Developed on a cohort of 722 patients and validated in 1006 patients with known emphysema, the algorithm calculates emphysema scores ranging from 0 to 1, which are categorized into None, Mild/Moderate, and Severe based on a weighted logistic regression calibrated to radiologist findings. Specifically, a score below 0.117^3^ corresponded to no emphysema, scores between 0.117^3^ and 0.215^3^ represented mild to moderate emphysema, and scores above 0.215^3^ indicated severe emphysema.

The algorithm’s primary utility lies in its ability to establish a clear progression of emphysema severity, moving from no emphysema to mild or moderate emphysema, and finally to severe emphysema. This approach prioritizes the consistency and clinical relevance of the ordered classification over the precise numerical cutoff points. While the cutoff thresholds were calibrated based on radiology reports, the emphasis remains on capturing the gradual increase in severity to enhance the robustness and applicability of the algorithm. By integrating quantitative imaging criteria, the algorithm provides a scalable and reproducible method for assessing emphysema while enabling validation by radiologists. Since radiology reports from both institutions included in this study do not routinely quantify emphysema, this automated algorithm offers a practical and reliable solution for analyzing large-scale datasets. It facilitates radiologist-grade emphysema classification while significantly reducing the time and resources required for manual assessment.

### Variable selection & logistic regression model

A forward selection method using Akaike information criterion (AIC) using the ‘MASS’ package (version 7.3–58.1) in R statistical software (version 4.2.2) was used to select the combination of variables that would result in the most predictive model [25, 26]. These variables were used to build a logistic regression model in R to predict cases of chest tube placement. The model was developed with data from UCSF and validated on data from ZSFG, an external hospital, using bootstrapping and ROC (receiver operating characteristic) curve analysis.

### Nomogram and risk calculator

A nomogram was built from the logistic regression model using RStudio and the ‘rms’ package (version 6.3–0) [27]. A risk calculator was also built and published with R’s ‘shiny’ website package (version 1.7.3) using the previously developed logistic regression model [28].

### Statistical analyses

Statistical analysis was performed using both R and Python statistical functions. Continuous variables were represented as means ± standard deviations. The association between variables was assessed using Fisher’s exact tests for binary variables, and with a logistic regression for continuous variables. Models were evaluated with bootstrapped calibration curves and Harrell’s C-index. The significance level was set to 0.05. Variables were assessed in Python 3.8.8 using Pandas (version 1.2.4), Numpy (version 1.19.5), and SciPy (version 1.6.2) [29–31].

## Results

### Study population

A total of 2,512 patients who underwent CT-guided lung biopsy were included in the study, after excluding those with missing imaging and report as well as those with incomplete scans. Of these, 157 (6.7%) developed pneumothorax requiring chest tube as a complication of the procedure, with 1899 patients and 114 complications being from UCSF and 613 patients and 43 complications being from ZSFG. The mean age of the UCSF cohort was 64.4 ± 14.2 years and 64.6 ± 10.4 years for the ZSFG validation cohort, and 52% (988/1899) and 59.0% (362/613) were male for the UCSF and ZSFG datasets, respectively. Approximately half of biopsies (UCSF: 53.23% (1011/1899), ZSFG: 47.47% (291/613)) were performed in the prone position. Further initial covariate distributions are presented in Table 1.

**Table 1:** Summary of initial covariates in both datasets

	Chest Tube in UCSF Data (n = 1899)			Chest Tube in ZSFG Data (n = 613)		
Risk Factor	No (n = 1785)	Yes (n = 114)	P-value	No (n = 570)	Yes (n = 43)	P-value
Age (years)	64.1 ± 14.3	68.2 ± 11.6		64.5 ± 10.3	65.8 ± 11.0	
< 40	124 (6.94%)	2 (1.75%)	.03 *	12 (2.10%)	0 (0%)	1
Between 40 & 60	458 (25.65%)	22 (19.29%)	.14	165 (25.94%)	11 (25.58%)	.72
> 60	1203 (67.39%)	90 (78.94%)	.009 *	393 (68.94%)	32 (74.41%)	.49
Male	922 (51.65%)	66 (57.89%)	.20	336 (58.94%)	26 (60.46%)	.87
Non-Prone Position	812 (45.49%)	76 (66.66%)	.01 *	283 (49.64%)	8 (18.60%)	<.001 *
Left Lobe Nodule	847 (47.45%)	56 (49.12%)	.77	246 (43.15%)	21 (48.83%)	.52
Emphysema Score	0.05 ± 0.16	0.07 ± 0.17	.26	0.08 ± 0.20	0.03 ± 0.05	.10
Emphysema Extent						
None	913 (51.14%)	42 (36.84%)	.01 *	318 (55.78%)	12 (27.90%)	.038 *
Mild/Moderate	355 (19.88%)	21 (19.42%)	.80	82 (14.38%)	12 (27.90%)	.026 *
Severe	517 (28.96%)	51 (44.73%)	<.001 *	170 (29.82%)	19 (44.18%)	.059

*Significant at an alpha level of 0.05

Description: Demographics table showing the numeric summary of initial covariates. Continuous variables are shown as mean ± standard deviation. P-values are from Fisher exact tests on binary variables and logistic regression for continuous variables

### Preprocessing

**Fig. 1:**
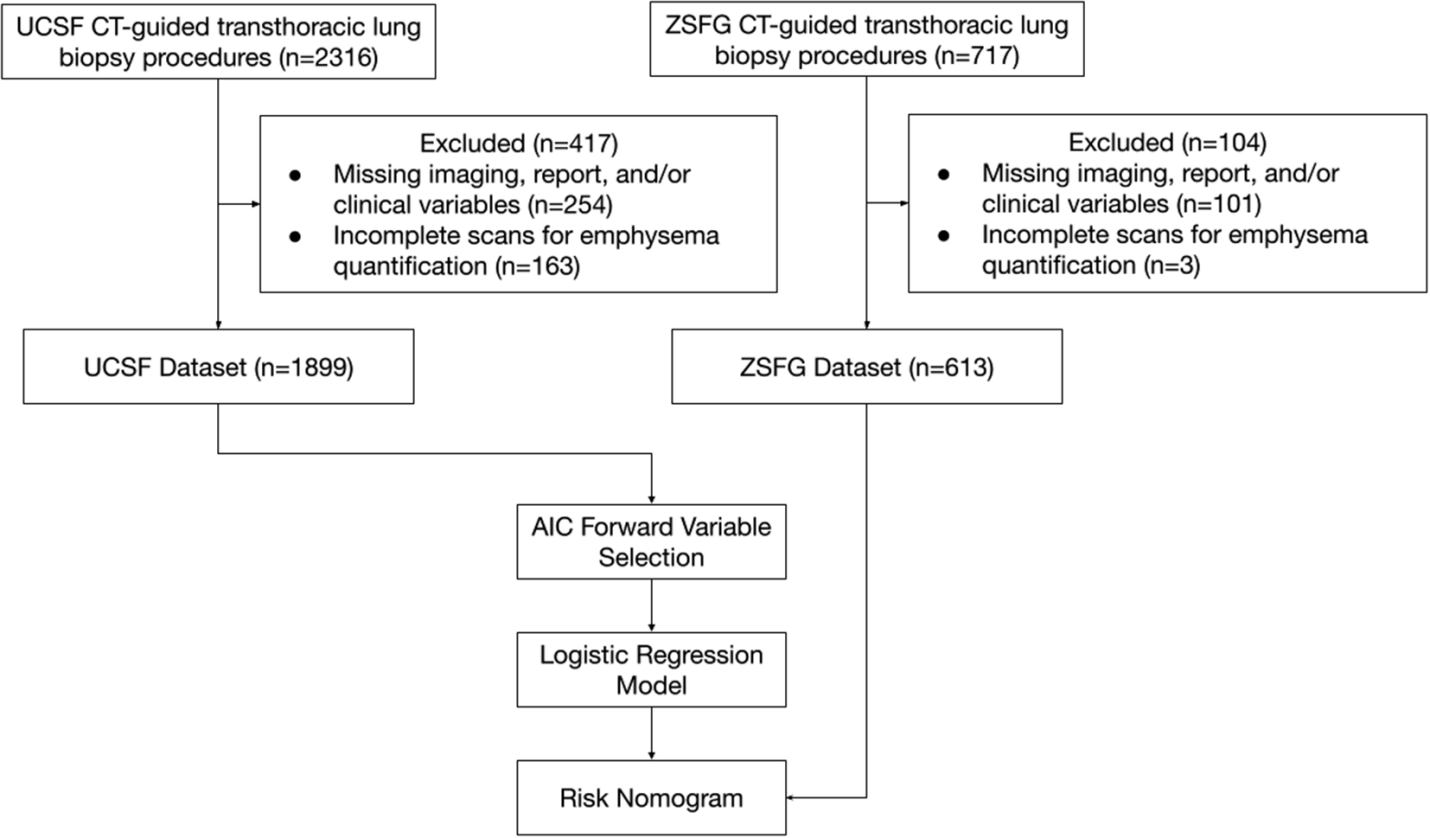
Flow diagram shows patients who underwent CT-guided transthoracic lung biopsy procedures. AIC = Akaike information criterion

The flow diagram in Fig. 1 demonstrates the dataset creation and preprocessing steps. Not imputable cases with missing nodule location, patient position, and/or gender, totaled to 254 in the training set, and 101 in the validation set, and were ultimately dropped. The predominant patient positions were prone and supine, so left lateral decubitus and right lateral decubitus positions (each accounting for less than 2.7% of the dataset) were combined with supine into the non-prone category.

### Quantitative emphysema algorithm

The emphysema algorithm successfully completed its analysis on all but 163 cases in the training set and 3 in the validation set. These cases were dropped per the exclusion criteria. The percent of patients with none, mild/moderate, and severe emphysema were 50.28% (955/1899), 19.79% (376/1899), and 29.91% (568 /1899) in the training dataset. In the validation set, this corresponded to 53.83% (330/613), 15.33% (94/613), and 30.83% (189/613).

### Variable selection & logistic regression model

Through multivariable stepwise forward AIC, the following variables were considered in the starting model: male, nodule location left lung, prone position, non-prone position, no emphysema, mild to moderate emphysema, severe emphysema, age less than 40, age between 40 and 60, age over 60, and emphysema score. Age over 60 (OR 1.8 [95% CI: 1.1–2.9], P =.01), non-prone patient position (OR 2.4 [1.6–3.7], P < 0.001), and severe emphysema (OR 1.9 [1.3–2.9], P < 0.001) were found to be the best combined predictors of complication. These variables were then fed into the logistic regression model, which achieved a Harrell’s C-index of 0.664 and 0.715 for the training and validation datasets, respectively. A summary of these multivariable findings is presented in Table 2.

**Table 2:** Multivariable Analysis after Model Selection

Feature	Odds Ratio	[95% CI]	P-value	Coefficient
Intercept	0.01	[0.01, 0.03]	<.001*	−3.95
Non-Prone Position	2.4	[1.6, 3.7]	<.001*	0.91
Severe Emphysema	1.9	[1.3, 2.9]	<.001*	0.69
Age > 60	1.8	[1.1, 2.9]	.01*	0.59

*Significant at p-level of 0.05

Description: Table presents the results from the final logistic regression multivariable analysis performed on the training data (n = 1899). Final variables were selected through Akaike Information Criterion (AIC) forward variable selection from the following factors: male, left lung nodule, age under 40, age between 40 and 60, age over 60, continuous emphysema score, no emphysema, mild/moderate emphysema, severe emphysema, and non-prone position. Emphysema categories were determined by thresholds on values from a quantitative emphysema algorithm. Non-prone position, severe emphysema, and age over 60 provided the strongest model

There were 34 cases (34/1899; 1.79%) within the UCSF dataset and 11 cases (11/613; 1.79%) within the ZSFG dataset that were false negatives. There were 809 (809/1899; 42.60%) and 297 (297/613; 48.45%) false positives in the UCSF and ZSFG datasets, respectively. Supplemental Materials 5 and 6 provide a summary of the most notable misclassified cases from both datasets.

**Fig. 2:**
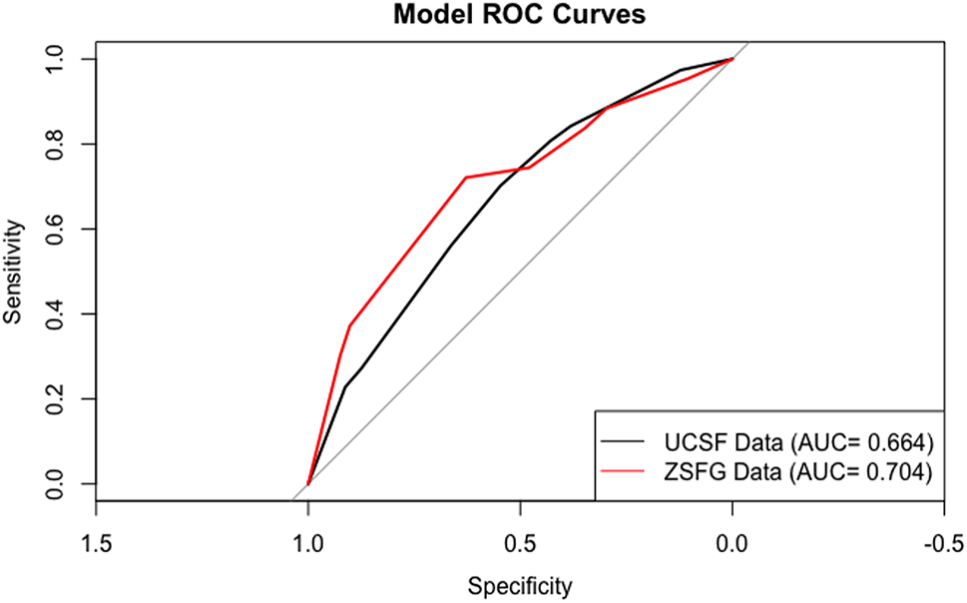
ROC Analysis shows curves for both training and validation datasets, with the validation curve being higher than the training curve

The model’s calibration was confirmed on the bootstrapped calibration plot, which can be found in Supplemental Material 1, as well as with calibration curves, which can be found in Supplemental Material 2. An ROC curve can be found in Fig. 2. The area under the curve (AUCROC) was 0.664 and 0.704 for UCSF and ZSFG, respectively. The sensitivity and specificity for UCSF was 0.966 and 0.089, respectively, and was 0.961 and 0.097 for ZSFG, respectively.

### Nomogram and risk calculator

**Fig. 3:**
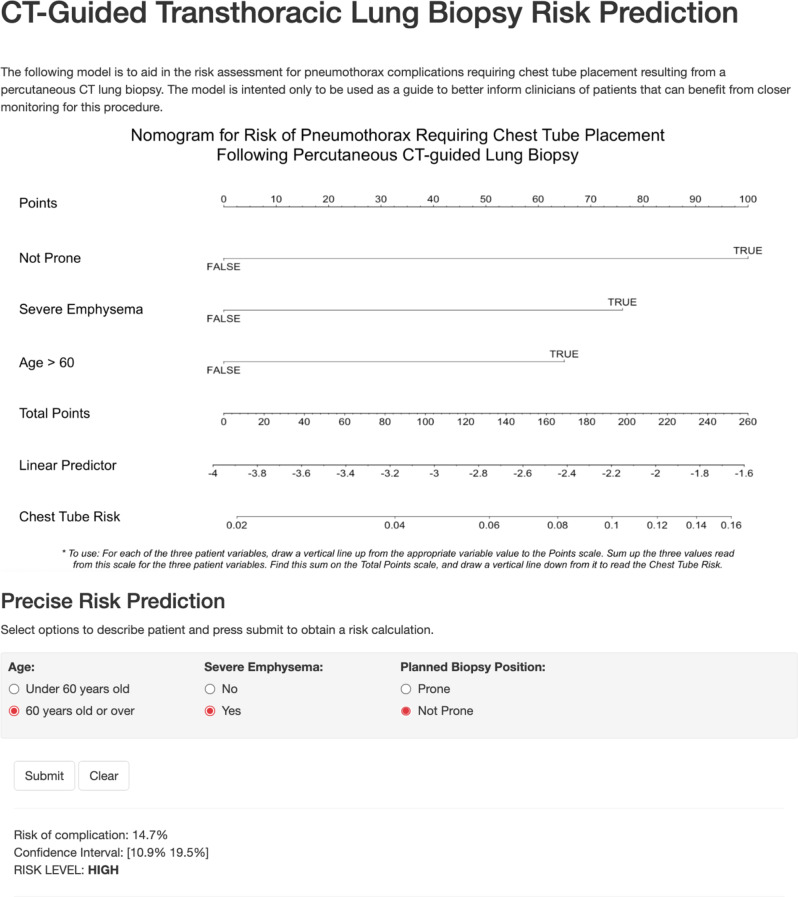
Screenshot from risk calculator website showing nomogram and risk calculator, with an example prediction. Website is publicly available at https://bit.ly/3n9Yu38

**Fig. 4:**
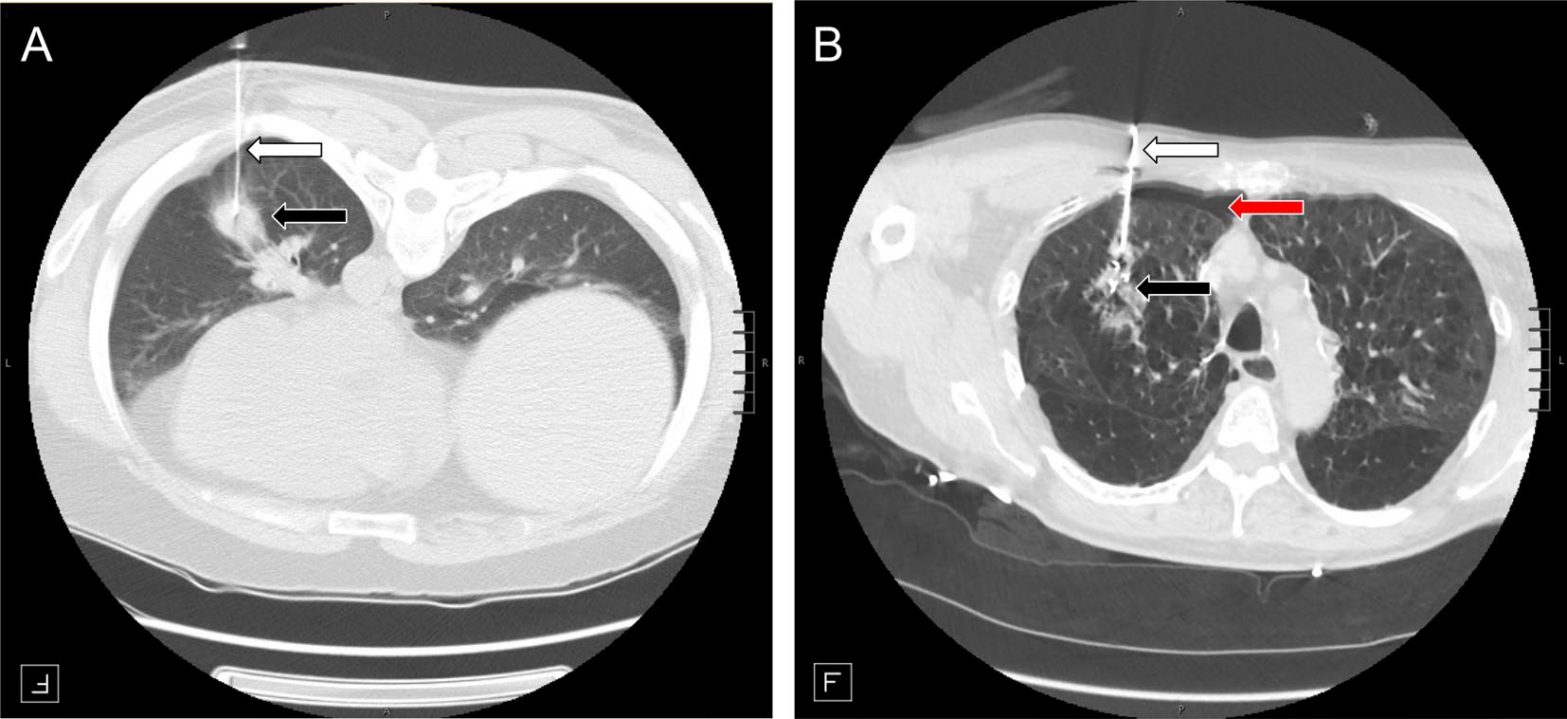
Example cases. CT scans from CT-guided transthoracic lung biopsy in two patients. Biopsy needle (white arrow) and the nodule biopsied (black arrow) are shown. **A)** Patient is a male in their 40s presenting with no emphysema (as determined by quantitative emphysema algorithm), lying in the prone position, with a nodule in the left lower lobe. Our risk nomogram and calculator predicted this patient to have low risk of pneumothorax requiring chest tube (1.86% [1.13% - 3.13%]). The patient post biopsy had a tiny pneumothorax and did not need chest tube placement. **B)** Patient is a male in their 60s with severe emphysema (as determined by quantitative emphysema algorithm), lying in the supine position, and with a nodule in the right middle lobe. Our risk nomogram and calculator predicted this patient to have high risk (14.70% [10.90% - 19.50%]). The patient developed intraprocedural pneumothorax (red arrow) which grew into a large pneumothorax and chest pain necessitating a chest tube placement

Based on the logistic regression model, a nomogram and a risk calculator (https://bit.ly/3n9Yu38) were developed to predict the risk of pneumothorax with a threshold for positivity at 0.06, which was the average positivity rate for the datasets. Figure 3 displays the nomogram and presents a screenshot of the website. The risk calculator classified patients into low, moderate, and high-risk groups based on their emphysema extent, age, and position during the biopsy. The thresholds for these categories were based on the median (4.562%) and the third quartile (7.953%) of the risks of the UCSF’s patients as determined by the model. This corresponded to the proportion of patients in the low, moderate, and high-risk groups for UCSF's data to be 41.4% (788/1899), 23.4% (445/1899), and 35.0% (666/1899), respectively. This proportion was generally maintained in the validation set with the proportion of patients in the low, moderate, and high-risk groups being 33.4% (205/613), 26.9% (165/613), and 39.64% (243/613). Generally, ZSFG had patients with higher risk than UCSF. A distribution of risk predictions for both datasets is graphed in Supplemental Material 3, and a demonstration of risk groups can be found in Table 3. Figure 4 presents example cases and predictions.

**Table 3:** Risk Groups Stratification

Age > 60	Severe Emphysema	Not Prone	Risk Percent [95% CI]	Risk Group
			1.89% [1.13% - 3.13%]	LOW
✓			3.36% [2.31% - 4.85%]	LOW
	✓		3.70% [2.19% - 6.20%]	LOW
		✓	4.56% [2.94% - 7.01%]	LOW
✓	✓		6.50% [4.47% - 9.35%]	MODERATE
✓		✓	7.95% [5.97% - 10.50%]	MODERATE
	✓	✓	8.73% [5.52% - 13.50%]	HIGH
✓	✓	✓	14.70% [10.90% − 19.50%]	HIGH

Description: This table presents the predicted risk and corresponding risk group for various combinations of variables in the context of pneumothorax requiring chest tube

Error analysis of false negative cases by thoracic radiologists demonstrated no identifiable systematic biases.

## Discussion

Our study investigated factors predictive of pneumothorax requiring chest tube placement following CT-guided lung biopsy, utilizing a large dataset (>2000 cases), a quantitative emphysema algorithm, and an external validation cohort. To enhance usability, the model was simplified to include non-prone position, severe emphysema, and age over 60 as key predictors. These variables were jointly used to categorize pneumothorax risk and develop an online risk nomogram. The risk calculator achieved Harrell’s C-index values of 0.664 and 0.715 in the testing and validation cohorts, respectively, indicating modest discriminatory ability.

Our risk factor findings are primarily consistent with several previous studies, specifically in regards to non-prone position [22, 23, 32] and older age [1, 3, 14, 16]. Geraghty et al. similarly found that age discretized at 60 years was statistically significant, whereas age as a continuous variable was not. Additionally, patient positioning, particularly non-prone positions such as supine and lateral, has been consistently associated with increased risk in studies by Nakamura et al., Takeshita et al., and Ruud et al. Regarding emphysema, our results support earlier research suggesting that severe emphysema is a strong predictor of complications requiring chest tube placement. Studies by Kazerooni et al. and Laurent et al. also linked severe obstructive lung disease with increased risk, though their findings were limited by smaller sample sizes and lack of external validation—gaps addressed by our study.

Our 3×3×3 kernel-based quantitative emphysema algorithm, calibrated against radiologists’ assessments and followed by thresholding to classify into three severity levels (none, mild/moderate, and severe), contributed significantly to the model’s reliability and predictive power. Previous studies that leveraged quantitative emphysema classification, such as those using Hounsfield Unit (HU) thresholds between − 950 and − 850 HU to calculate percent emphysema in the lungs, have demonstrated limitations. In our earlier analyses, this simplified approach was found to overestimate the extent of emphysema, making it more prone to inaccuracies.

Theilig et al. found a significant association between quantitative emphysema and pneumothorax risk, whereas Chami et al. and Lendeckel et al. did not observe such associations in their studies. Notably, these prior studies were limited by sample sizes of fewer than 400 biopsy cases. Our study, utilizing a larger dataset and external validation, provides additional evidence that quantitative emphysema scores classified into three-point scales are strongly associated with pneumothorax risk. The algorithm’s ordinal classification, designed to capture a clear progression of emphysema severity, enhances its flexibility and scalability, enabling both quantitative analysis and qualitative assessments by radiologists when necessary.

While the nomogram was designed for clinical usability, its performance is not without limitations. The modest AUCROC values (0.664 and 0.715) underscore the need for refinement and highlight the influence of unmeasured or unrecorded variables on pneumothorax risk suggest room for refinement and reflect the influence of unmeasured variables on pneumothorax risk. A limitation of this study lies in the retrospective nature of the data and the variability in chest tube placement decisions. The indication for chest tube insertion following biopsy-related pneumothorax is influenced not only by the radiologic extent of the pneumothorax but also by clinical factors such as patient symptoms, baseline lung function, and institutional or provider-specific management protocols. As such, the outcome of “pneumothorax requiring chest tube” may exhibit some heterogeneity in clinical scenarios, potentially influencing the model’s discriminatory performance. Additionally, the use of standardized radiology reports limited the level of procedural detail available, further constraining our ability to fully capture relevant variables. Future studies that incorporate prospective data collection and standardized documentation of procedural and clinical criteria, such as chest tube placement, will be essential to improve outcome specificity and support further refinement of the model’s predictive capabilities.

The inclusion of data from two hospitals with distinct patient populations and procedural protocols enhances the generalizability of our findings. ZSFG, as a county safety-net hospital, serves a sicker patient population with more advanced disease compared to UCSF. While this diversity reflects real-world variability, it also introduces potential confounders, such as differences in case severity and procedural outcomes. Minor procedural differences between the two hospitals, such as sedation practices and post-biopsy patching techniques, likely influenced outcomes but also provide a realistic validation of the model. Future research should include stratified analyses to explore the impact of these institutional differences on model performance.

This tool provides clinicians with additional information to support patient management decisions, such as considering referral to tertiary medical centers or exploring alternative biopsy approaches (e.g., bronchoscopic biopsy) to reduce risk. However, its modest discriminatory ability and retrospective design limit its readiness for widespread clinical implementation. Future studies should prioritize additional external validation across diverse hospital sites, further calibration of the model, and the integration of advanced imaging techniques to enhance prediction accuracy.

In conclusion, we propose a simplified risk nomogram for substantial pneumothorax following lung biopsy, integrating older age, emphysema severity, and patient position. While the model shows promise, further validation and refinement are essential to improve its predictive performance and clinical applicability.

## Electronic supplementary material

Below is the link to the electronic supplementary material.

Supplementary Material 1

Supplementary Material 2

Supplementary Material 3

Supplementary Material 4

Supplementary Material 5

Supplementary Material 6

## Data Availability

The data from this project can be shared with researchers per data use agreement and approval from UCSF.
