# Supplementary figures and images for: Development and validation of a risk nomogram predicting pneumothorax requiring chest tube placement post-percutaneous CT-guided lung biopsy
Source: BMC Med Imaging. 2025 Jul 1;25:220. doi: 10.1186/s12880-025-01794-y (PMC12211772; doi:10.1186/s12880-025-01794-y)

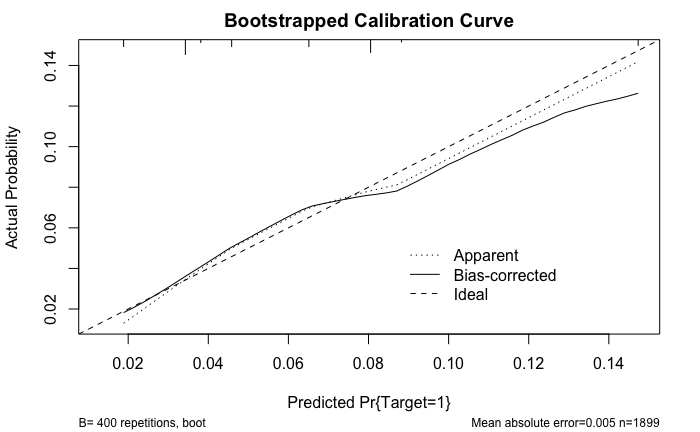

Supplement: Supplementary file 1 — Supplementary Material 1 [file 12880_2025_1794_MOESM1_ESM.png]

Calibration plot for UCSF data

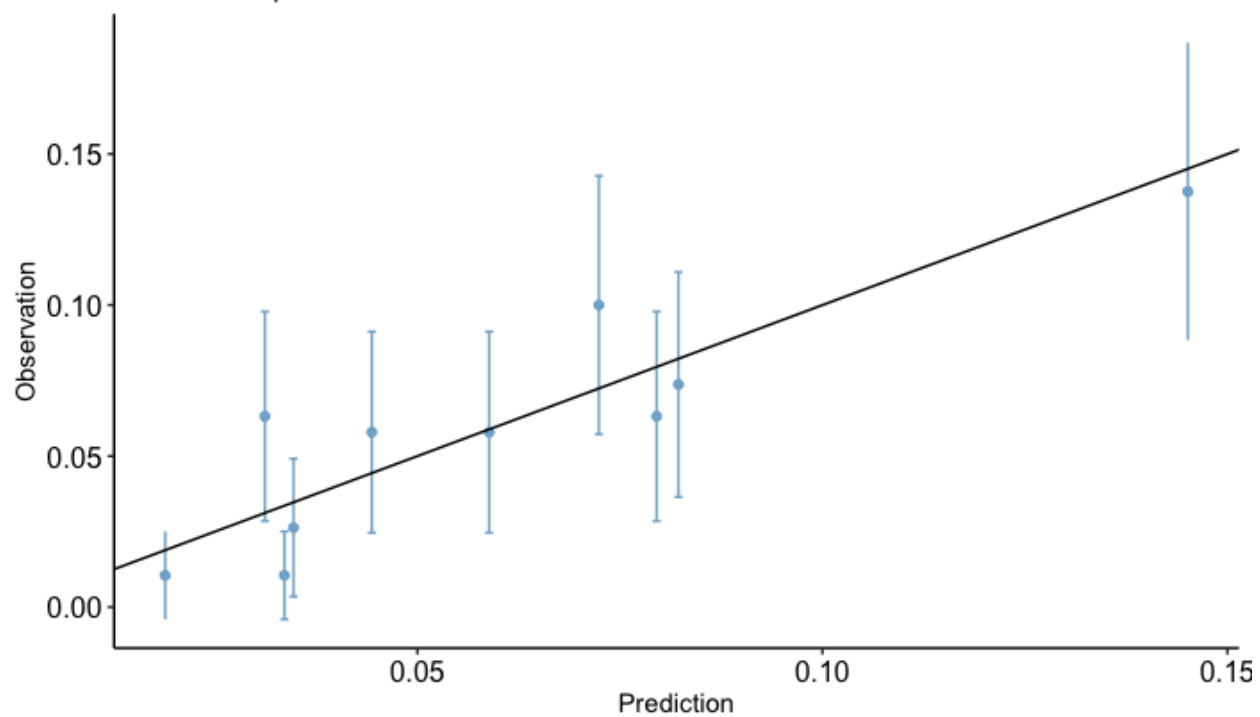

Calibration plot for ZSFG data

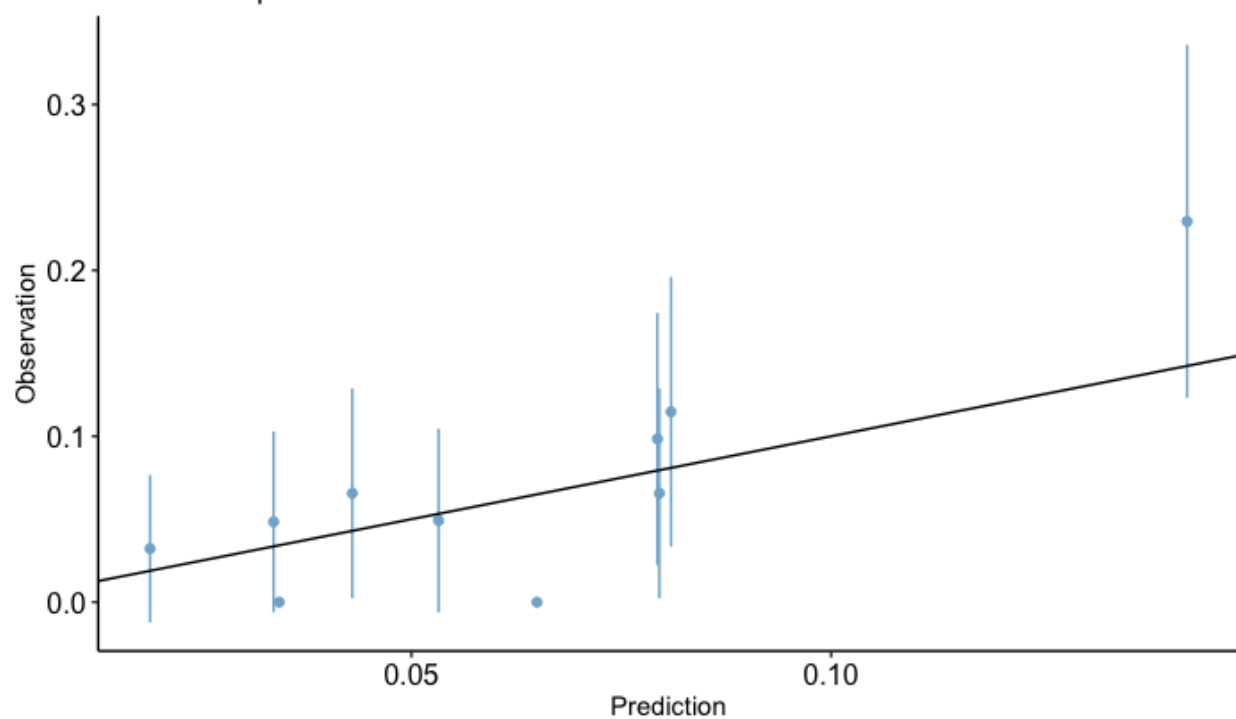

Supplement: Supplementary file 2 — Supplementary Material 2 [file 12880_2025_1794_MOESM2_ESM.pdf]

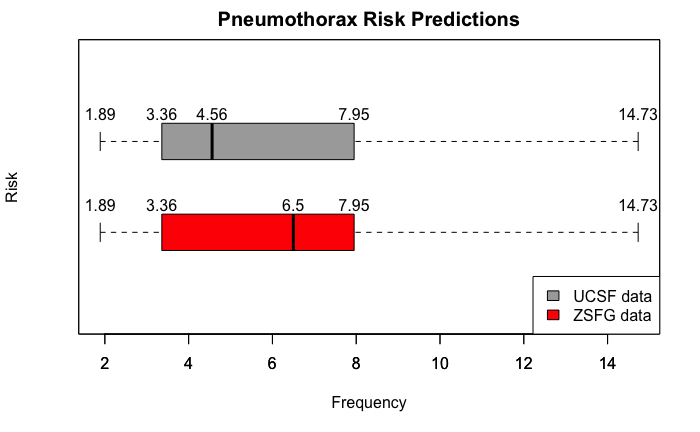

Supplement: Supplementary file 3 — Supplementary Material 3 [file 12880_2025_1794_MOESM3_ESM.png]
