# Supplementary material for: Development and validation of a risk nomogram predicting pneumothorax requiring chest tube placement post-percutaneous CT-guided lung biopsy
Source: BMC Med Imaging. 2025 Jul 1;25:220. doi: 10.1186/s12880-025-01794-y (PMC12211772; doi:10.1186/s12880-025-01794-y)
Supplement: Supplementary file 4 — Supplementary Material 4 [file 12880_2025_1794_MOESM4_ESM.docx]

**SUPPLEMENTARY FIGURES AND TABLES**

**Supplemental Material 1:** Figure shows bootstrapped calibration plot for the training dataset, where ‘Target’ refers to cases of pneumothorax requiring chest tube. 400 repetitions were run to create apparent, bias-corrected, and ideal model lines. A perfect model has apparent and bias-correct lines closer to the ideal line. Based on this calibration plot, our model appears to be well calibrated, with a mean absolute error of 0.005.

**Supplemental Material 2:** Figure shows calibration plots for both cohorts.

**Supplemental Material 3:** Figure shows distribution of risk scores for both training and validation datasets. Figure shows both datasets have the same range and quartiles of predictions, but with different medians. The validation dataset had the higher median.

**Supplemental Material 5:** Error Analysis on UCSF Cohort

This table summarizes the false negative and false positive cases for the UCSF cohort. The first represents the most extreme false positive, the second row shows the most extreme false negative, and the last row shows an additional false negative subset of cases. UCSF had 34 false negative cases. There were 809 false positive cases in UCSF dataset.

**Supplemental Material 6:** Error Analysis on ZSFG Cohort

This table summarizes the false negative and false positive cases for the ZSFG cohort. The first represents the most extreme false positive, the second row shows the most extreme false negative, and the last row shows an additional false negative subset of cases. ZSFG had 11 false negative cases. There were 297 false positive cases in the ZSFG dataset.
