# Supplementary material for: Development and validation of a risk nomogram predicting pneumothorax requiring chest tube placement post-percutaneous CT-guided lung biopsy
Source: BMC Med Imaging. 2025 Jul 1;25:220. doi: 10.1186/s12880-025-01794-y (PMC12211772; doi:10.1186/s12880-025-01794-y)
Supplement: Supplementary file 5 — Supplementary Material 5 [file 12880_2025_1794_MOESM5_ESM.docx]

**Error Analysis on UCSF Cohort**

| **Number of Cases** | **Severe Emphysema** | **Non-Prone** | **Age > 60** | **Risk**  **Score** | **Chest Tube**  **Predicted** | **Chest Tube Needed** |
| --- | --- | --- | --- | --- | --- | --- |
| 207 | ✓ |  | ✓ | 6.49% | Yes | No |
| 12 |  | ✓ |  | 4.56% | No | Yes |
| 2 |  |  |  | 1.88% | No | Yes |
